# Characteristics of a novel citizen rescue system for out-of-hospital cardiac arrest in the Dutch province of Limburg: relation to incidence and survival

**DOI:** 10.1007/s12471-018-1215-0

**Published:** 2018-12-17

**Authors:** R. W. M. Pijls, P. J. Nelemans, B. M. Rahel, A. P. M. Gorgels

**Affiliations:** 1 Department of Cardiology, CAPHRI School for Public Health and Primary Care, Maastricht University Medical Centre+, Maastricht, The Netherlands; 2 Department of Epidemiology, CAPHRI School for Public Health and Primary Care, Maastricht University Medical Centre+, Maastricht, The Netherlands; 3 Department of Cardiology, Viecuri Medical Centre for Northern Limburg, Venlo, The Netherlands

**Keywords:** Out-of-hospital circulatory arrest, Incidence, Survival, Citizen rescuer

## Abstract

**Background:**

We evaluated the characteristics of a novel text message system notifying citizen rescuers in cases of out-of-hospital circulatory arrest (OHCA) in the Dutch province of Limburg, including their relation to incidence and survival.

**Methods and results:**

The study area comprised 2,153 km^2^ (831 mi^2^) with 1.12 mio. inhabitants. During the 2‑year study period approximately 9,000 volunteers were registered, about 60% male, 59% with no experience in actual resuscitation, and 27.4% healthcare professionals. The system was not activated in 557 of 1,085 (51.3%) OHCAs, frequently because there was no resuscitation setting present yet at the time of the emergency call. Rescuers were notified on 1,076 occasions, with no resuscitation setting being present in 548 of 1,076 (50.9%) notifications. OHCA incidence rates were 67 per 100,000 inhabitants per year, 95 per 100,000 men and 39 per 100,000 women standardised for age with the European Standard Population. The mean number of notifications per volunteer was 1.3 times per year. Higher volunteer density was related to increased survival if at least one volunteer attended the cardiac arrest. If the density exceeded 0.75%, survival increased to 34.8% compared to 20.6% at a density below 0.25%.

**Conclusion:**

In about half of OHCAs needing resuscitation the system was activated and in approximately half of the notifications resuscitation proved to be justified. Volunteers are notified 1.3 times per year on average. Survival was related to volunteer density, suggesting that further improvement can be achieved by increasing the number of citizen rescuers.

## What’s new?

- Dutch incidence (crude and standardised for age) of out-of-hospital circulatory arrest (OHCA).
- In about half of the OHCAs needing resuscitation the alert system is activated.
- Resuscitation proved to be justified in about half of the notifications.
- Volunteers are notified 1.3 times per year on average.
- Survival following OHCA is related to volunteer density.

## Introduction

In the Netherlands a citizen volunteer alert system was launched to be activated in cases of out-of-hospital circulatory arrest (OHCA). This zip-code-based system substantially contributes to survival following OHCA with a cardiac cause [1], specifically in the home [2], where most OHCAs occur.

The activation process has not been described to date. The present study was designed to investigate how frequently the system is activated in cases of OHCA and to identify reasons why the notification system was not activated. We also investigated the mean notification rate per volunteer and if volunteer density has an influence on survival.

## Methods

Included in the study were witnessed and unwitnessed OHCAs in patients of all ages with sudden loss of vital signs, where the ambulance service was notified. Cases occurring in the terminal phase of a disease were excluded.

### Setting

Data were used from an Utstein-based [3] registry comprising all cases of OHCA in the Dutch province of Limburg during the period April 2012 to April 2014, covering an area of 2,153 km^2^ (831 mi^2^) with 1.12 mio. inhabitants (MUMC+ approved project number 114029).

### Resuscitation volunteer network

The basic professional procedure during an OHCA in the Netherlands consists of dispatching two ambulances to the resuscitation location. Additionally, a text message (TM) alert system can be activated which in turn will notify certified volunteers trained in providing basic life support (BLS) and the use of an automated external defibrillator (AED). To determine which volunteers and AEDs are possibly closest to the victim within a 1-km (0.62-mi) radius, the system uses the zip codes of the victim, registered volunteers and AEDs. The system aims to select and send a TM to volunteers directing them in a 1:2 fashion either immediately to the victim or to collect a system-registered AED first. To establish an adequate but not excessive number of citizen rescuers, a maximum of 30 volunteers are notified.

During the study, 17 (including both dispatch centres in Limburg) of the 24 dispatch centres in the Netherlands were using the system consisting of approximately 66,500 registered volunteers (about 9,000 volunteers in the Dutch province of Limburg).

### Data collection

The following sources were used to retrieve data: (1) the dispatch centres at Limburg North and South; (2) their corresponding emergency medical services (EMS); (3) TM-alert system organisation (*Hartslagnu*); (4) notified volunteers; (5) the six hospitals in Limburg; and (6) AED providers.

Data retrieved from the dispatch centres and corresponding EMS consisted of notification time, ambulance departure time and time of arrival at the location, survival at hospital discharge, and information about the resuscitation scenario (e. g. whether the OHCA was witnessed or not, whether BLS was started and by whom, and whether citizen rescuers attended the OHCA). Alert system information such as the time the TM was sent, the number of volunteers notified and AEDs, and type of notification (immediately start BLS or first obtain an AED) was acquired from the TM-alert system organisation. A questionnaire was sent to all notified volunteers in order to obtain information about their attendance and, if applicable, about details of the cardiopulmonary resuscitation (CPR) scenario. The patient’s medical history and post-resuscitation treatment were obtained from the six hospitals in Limburg.

### Statistical analysis

Data of all resuscitated and non-resuscitated OHCA victims in the 2‑year study period were used to calculate crude, age-standardised and age- and sex-specific incidence rate per 100,000 inhabitants per year. Information on the number of men and women per 5‑year age category in the province of Limburg in 2013 were obtained from Statistics Netherlands (CBS). The European Standard Population was used to calculate age-standardised incidence rates. Categorical variables were described as absolute numbers and percentages, and continuous variables as means with standard deviation or medians with interquartile range.

The statistical software package of SPSS (SPSS for Windows, version 22.0, SPSS Inc, Chicago, IL) was used to analyse the data.

## Results

During the study period, 1,546 OHCAs including 461 victims with prolonged death were recorded. Resuscitation was indicated in 1,085 cases who were still alive at arrival of one or more volunteers or the ambulance.

### Characteristics of citizen rescuers

During the study period more than 9,000 volunteers were registered in Limburg. About 60% were male, around 59% had no previous experience in performing resuscitation, 27.4% were healthcare professionals of whom 51.5% had a nursing background, 32% a paramedical profession, 6.4% being physicians and around 10% medical students.

### Incidence of OHCA

Based on a total of 1,546 OHCAs and prolonged deaths, crude incidence was 69 per 100,000 inhabitants, 94 per 100,000 men and 44 per 100,000 women.

**Fig. 1:**
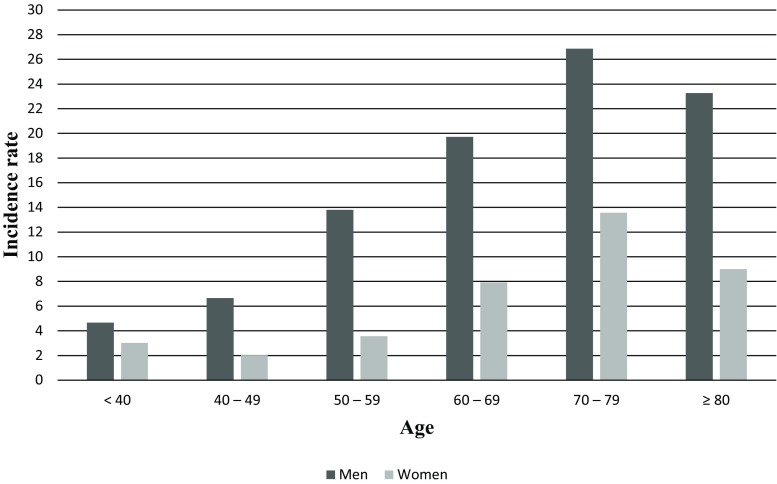
Sex- and age-specific incidence of OHCAs per 100,000 inhabitants *OHCA* out-of-hospital circulatory arrest

Fig. 1 depicts the age-specific incidence rates of OHCAs per 100,000 for both sexes. Incidence increased up to age group 70–79 and was consistently higher in men. After standardisation for age with the European Standard Population, incidence was 67 per 100,000 inhabitants, 95 per 100,000 men and 39 per 100,000 women.

### Coverage of the system of patients needing resuscitation

**Fig. 2:**
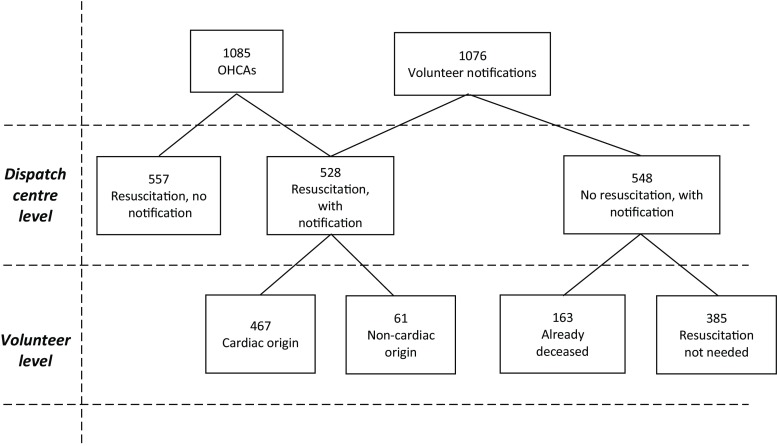
Flowchart of the 1,085 attempted resuscitations and 1,076 notifications in relation to the dispatch centre level and volunteer level. *OHCA* out-of-hospital circulatory arrest

A total of 1,085 OHCAs requiring resuscitation were recorded (Fig. 2). Volunteers were notified in 528 of 1,085 OHCAs (48.7%), 467 (88.4%) with a cardiac and 61 (11.6%) with a non-cardiac origin. The reasons for not activating the system were evaluated in a sample of 351 of these 557/1,085 cases (Tab. 1).

**Table 1:** Reasons for not activating the rescue system

Reason	n (%)
No OHCA setting yet	168 (47.9)
Zip code not known	54 (15.4)
(Semi) medical help already at location	31 (8.8)
Onsite AED and rescuers at location	30 (8.5)
Bad prognosis evident	14 (4.0)
OHCA not recognised by dispatcher	3 (0.9)
Patient with known DNR policy	3 (0.9)
Other reasons^a^	14 (4.0)
Reason unknown	34 (9.7)

*OHCA* out-of-hospital circulatory arrest, *AED* automated external defibrillator, *DNR* do not resuscitate

^a^Shooting incident, inaccessible area etc

In 47.9% a condition preceding OHCA, such as chest pain or dyspnoea, evoked the emergency call. So at the moment of the OHCA the ambulance was already heading towards or present at the location. Other reasons were lack of zip-code information of the OHCA location (15.4%), medical help such as medical staff during a sports event (8.8%) or local rescuers and AED on site (8.5%) already present, evidently poor prognosis (4.0%), OHCA not recognised by the dispatcher (0.9%), known do-not-resuscitate (DNR) policy (0.9%), other reasons (unspecified) (4.0%), no reason identified (9.7%).

### Frequency of justified volunteer notifications

Volunteers were notified in 1,076 cases (Fig. 2). Of all notifications 528 (49.1%) concerned actual resuscitations (including DNR and in-ambulance OHCAs). The 548 non-resuscitation settings (Fig. 2) concerned reversible collapses (*n* = 385) due to insults, alcohol intoxication, vagal episodes, pulmonary insufficiency, cerebral accidents or terminal disease, or prolonged deaths (*n* = 163).

Based on 1,076 notifications (in the 2‑year study period) and a mean of 21.9 volunteers notified per case, annual notifications amounted to 11,782 [(1,076 × 21.9) / 2]. Given 9,000 available volunteers the mean number of annual notifications per volunteer is 1.30, half (0.65 per year) concerning actual resuscitations.

### Volunteer density and survival

**Fig. 3:**
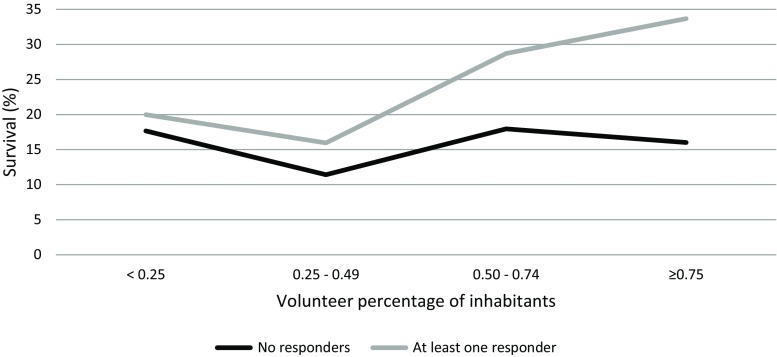
Gain in percentage survival at discharge from hospital according to volunteers per number of inhabitants

The 32 municipalities of the study region were categorised according to number of volunteers/number of inhabitants as: <0.25%, 0.25–0.49%, 0.50–0.74% and ≥0.75. To evaluate the effectiveness of the system in relation to volunteer density we performed an analysis using 422 cases with OHCA due to a cardiac cause where the system was activated. Patients with a DNR policy and/or cardiac arrest in the ambulance were not included in this analysis, hence the difference compared with the 467 cardiac cases in Fig. 2. Within each density category, we compared the percentage survival to hospital discharge when at least one responder attended versus no responder attending. In the latter group, at higher densities no increase in survival was found. When volunteers attended, percentage survival increased with higher volunteer density (Tab. 2; Fig. 3).

**Table 2:** Percentage survival, gain in survival and proportion of OHCAs with at least one responder according to volunteer density

Volunteer density (%)	Survival with no responders (%, *n*)	Survival with at least one responder (%, *n*)	Gain in survival (%)	OHCA with at least one responder (%, *n*)
<0.25	18.2 (6/33)	20.6 (7/34)	2.4	51 (34/67)
0.25–0.49	11.8 (4/34)	15.9 (11/69)	4.1	67 (69/103)
0.50–0.74	17.9 (7/39)	30.2 (29/96)	12.3	71 (96/135)
≥0.75	16.0 (4/25)	34.8 (32/92)	18.8	79 (92/117)

*OHCA* out-of-hospital circulatory arrest

## Discussion

### Main findings

In the Dutch province of Limburg with an age-standardised OHCA incidence of 67 per 100,000 inhabitants per year, resuscitation was attempted in 1,085 cases within a 2-year period. The system was not activated in 557 (51.3%) cases, frequently due to the absence of an OHCA setting at the time of the emergency call. Volunteers were notified in 1,076 cases, with 528 (49.1%) victims actually needing resuscitation. Annual notifications per volunteer amounted to a mean of 1.30, 0.65 concerning actual resuscitations. The highest increase in survival due to the system (from 16.0 to 34.8%) occurred if volunteer density exceeded 0.75%, underscoring its current and future impact if the number of volunteers further increases. Citizen rescuers frequently had no real-life CPR experience nor a medical background, suggesting a sound opportunity to improve the system if more healthcare professionals would apply.

### Incidence rates

We assessed the incidence rate of OHCA based on our prospective registry with a day-to-day assessment of cases as recorded by the ambulance personnel. This method is more accurate than a retrospective death-certificate-based methodology, which leads to an overestimation of OHCA rates [4]. In agreement with Utstein recommendations [3] and due to the difficulty in differentiating between OHCA of cardiac and non-cardiac origin in many registries [5], we included both (presumed) cardiac and non-cardiac causes. As a result of meticulous evaluation of the records from the paramedics and the hospitals, we were able to identify the cause of OHCA in many instances [6]. To assess incidence more accurately we included, in contrast to many studies [7], OHCA victims who were found dead unexpectedly and in whom resuscitation had not been attempted. Similar incidence rates were reported from another region in the Netherlands [8]. A study from the Amsterdam region [9] showed an incidence rate of 60 per 100,000 inhabitants compared to the crude incidence rate of 69 per 100,000 inhabitants found in our study. The larger number of younger inhabitants in the Amsterdam population might be an explanation for this difference. A previous study performed in the Maastricht area in the 1990s [10] reported a crude incidence rate of 100 per 100,000 inhabitants. This higher estimate is due to the inclusion of prolonged deaths reported by the general practitioners. In all age groups, the incidence rate in men was higher than that in women, in agreement with previous studies [4, 8–13] where OHCA rates in men were 2–3 times higher. Incidence peaked in the age group 70–79 years.

### Resuscitations

The system was developed to improve survival following cardiac arrest. The majority (88.4%) of the OHCAs for which the system was activated had a cardiac cause. The dispatch centre did not activate the system in 51.3% of OHCAs. The most frequent reason was the absence of a resuscitation setting at the time of the emergency call, or the fact that (semi) medical help was already at the location or close by. Being a zip-code-based system, no notification could be performed if the zip code was unavailable (15.4%). Currently, in addition to the zip-code system a GPS-based system is being introduced, making it possible to notify possible rescuers independent of their zip code. Because no zip code is required this novel development will likely lead to an increase of correct notifications, as it will be possible for the dispatcher to notify volunteers for whom no zip-code information is available when a cardiac arrest is clearly recognised.

### Notifications

Volunteers are notified once per year on average, indicating a low burden for the volunteer. In 50.9% volunteers were notified when no OHCA was present. This may imply that there is room for improvement as regards communication between the witness and the dispatcher as well as in the assessment of potential OHCAs by dispatchers.

### Volunteer density and survival

We observed a positive correlation between the density of citizen rescuers and percentage of survival to hospital discharge. These findings suggest that survival may even further increase with higher numbers of volunteers. This is in line with the recently formulated criteria by the Dutch Heart Foundation (*Hartstichting*) for so-called 6‑minute zones. In order to provide adequate help within 6 min an active notification system should be maintained with a high density of volunteers and AEDs. During the study period the number of volunteers in the study region increased from 9,000 to 11,000.

### Limitations

This study was performed in just one area in the Netherlands, questioning the generalisability of our findings. Recent data from the Dutch Heart Foundation suggest comparable incidence rates in other parts of the country but survival rates differing between 13 and 27% [14] with a mean of 23%. Although ambulance services act according to similar legislation and treatment protocols and volunteers are trained according to national guidelines, suggesting equal BLS/AED skills all over the country, these regional differences in survival rates could be due to differences in volunteer densities and/or a variety of other factors. It is very difficult, however, to assess retrospectively which variables might be responsible.

In addition to the TM notification system a GPS-based notification system was introduced and at the time of writing the number of citizen rescuers nationwide has increased substantially. These considerations stress the need for a continuing registration of the number of volunteers and system activations in relation to effectiveness, allowing rational adjustments of the further implementation of the zip-code and GPS-based systems.

## Conclusions

The system covers about half of the OHCAs needing resuscitation; approximately half of the notifications was an actual resuscitation. The average number of notifications is 1.30 per year per volunteer. The burden for citizen rescuers can be reduced because notification still carries a 50% chance of resuscitation not being required. The higher increase in survival to hospital discharge in areas with a higher volunteer density suggests that the effectiveness of the system could be further improved if more volunteers per 1,000 habitants were to become involved. These findings are important for further implementation of this citizen rescuer system within the community.
